# Tasserkidriluscf.americanus (Clitellata, Naididae) – A new record from Slovakia confirms the dissimilarity between the European and North American populations

**DOI:** 10.3897/BDJ.9.e72846

**Published:** 2021-10-18

**Authors:** Igor Kokavec

**Affiliations:** 1 Slovak Academy of Sciences, Bratislava, Slovakia Slovak Academy of Sciences Bratislava Slovakia

**Keywords:** Tubificinae, distribution, ecology, morphology, Oligochaeta, swamp

## Abstract

The main objective of this study is to present a new record of Tasserkidriluscf.americanus found in a channel near the Tešmak swamp in Slovakia (Central Europe) and to compare its morphological features and habitat requirements with those of populations occurring in North America and Europe. The new specimens are similar to those found in The Netherlands and Belgium, but dissimilar to previously reported North American material of *T.americanus*, reopening the question of whether the European form is a separate species. The European form has the penis sheaths approximately twice as long as and wider than the North American form and may inhabit slow-flowing or standing waters of a eutrophic character, which is in conflict with the current knowledge on the morphology and ecology of North American populations. Further investigation is necessary to solve the questions about the origin and taxonomic relationship of the European population to other populations.

## Introduction

At present, the genus *Tasserkidrilus* Holmquist, 1985 includes 14 freshwater species, all in the Holarctic Region (Table 1). Of all the species, 11 were recorded in the Palearctic Region, of which nine species are endemic to Lake Baikal and one species is endemic to the Kamchatka Peninsula. Despite high endemism of the genus, there are two species, *T.americanus* (Brinkhurst & Cook, 1966) and *T.kessleri* (Hrabě, 1962), which have a more scattered distributional pattern, occurring in several countries in both Eurasia and North America.

The first documentation regarding the genus *Tasserkidrilus* is *Tubifexkessleri*, which was described by Hrabě (1962), was based on an single, incomplete specimen found in Lake Onega (Russia). Morphological features make this species easily recognisable from other tubificines – some anterior ventral chaetae with intermediate teeth and the funnel- or cone-shaped, chitinous penis sheaths, shown in Fig. 4 below. At present, the latter is considered a diagnostic feature for the identification of the genus *Tasserkidrilus*, which was later accepted as the correct genus assignment for many species previously classified in *Tubifex* (Holmquist 1985). Moreover, the taxon*Tubifexkessleri* has included several subspecies in the past, which are currently considered as valid species of *Tasserkidrilus* (Table 1).

Brinkhurst and Cook (1966) found worms with similar features to Hrabě's specimen in the Great Lakes of North America, although they differed by the presence of long hair chaetae in all anterior segments. The penis sheaths had the same shape and dimensions; however, they were not always as broad as in Hrabě's specimen. The material was described by Brinkhurst & Cook (1966) as a subspecies, *Tubifexkessleriamericanus*, which was subsequently elevated to species and selected as the type species of a new genus,*Tasserkidrilus*, by Holmquist (1985). The information provided by Reynolds and Wetzel (2021), who represent *Aulodrilusamericanus* Brinkhurst & Cook, 1966 as the type species of *Tasserkidrilus*, is incorrect.

All North American records of *T.americanus* date back to studies carried out more than 30 years ago (see GBIF Secretariat (2021)). At present, new records outside the main distribution area are very rare; for example, van Haaren and Soors (2013) documented its occurrence in Europe for the first time. On the basis of the different morphology of the penis sheaths and the environmental requirements of the European population, when compared with North American populations, those authors stated that they were observing a new “form”. The main goal of the study is to present a possible new record of the species T.cf.americanus from Central Europe and to compare the new information with briefly updated literature data about the species from elsewhere in the world.

## Material and Methods

**Figure 1.**
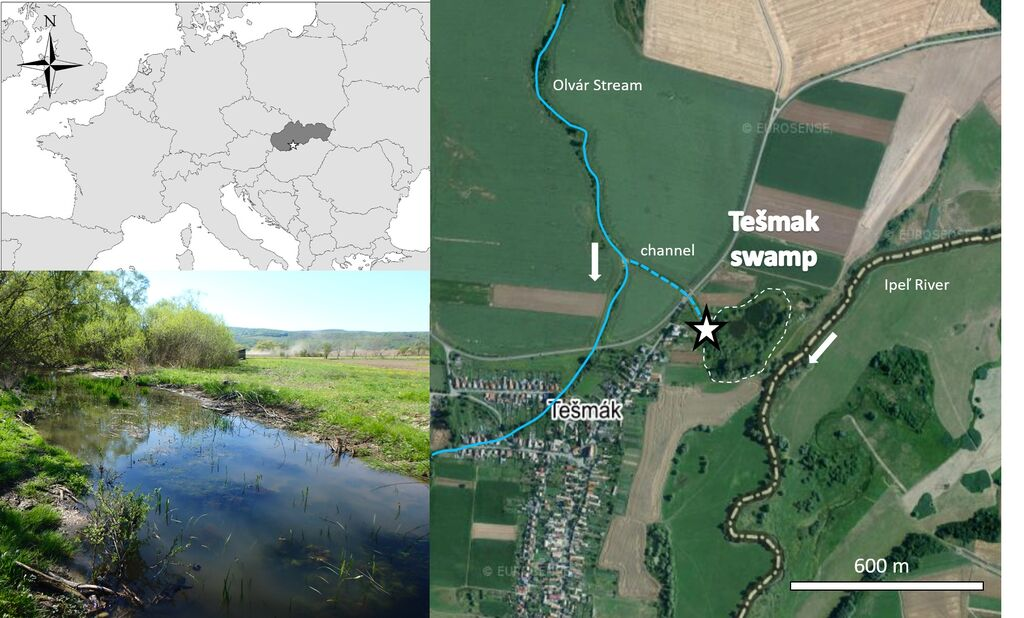
Map showing the locality of the Tešmak swamp in Slovakia and a photograph of the channel with the documented population of Tasserkidriluscf.americanus.

A macroinvertebrate sample was taken semi-quantitatively using a standardised hydrobiological mesh from a muddy bottom substrate, submerged plants and organic detritus in the channel flowing from the Olvár stream that supplies water to the Tešmak swamp in the southern part of Slovakia in April 2020 (Fig. 1, Suppl. material 1). The swamp and channel, which contribute water from the Olvár stream, are of a eutrophic character with seasonal water level fluctuations. The sampled material was fixed in a 4% formaldehyde solution in plastic sample containers. In the laboratory, the macroinvertebrates were sorted and preserved in 70% ethyl alcohol. Prior to identification, aquatic oligochaetes were processed through ethyl alcohol and clove oil to dehydrate and clear their bodies in order to study their internal tissues and organs. Subsequently, they were fixed in Canada Balsam as permanent mounts and kept in a dryer set at 40°C for four days. Species were identified using the binocular microscope Leica DMLB and the determination keys by Hrabě (1979), Kasprzak (1981), Timm (2009) and van Haaren and Soors (2013).

## Results

**Figure 2.**
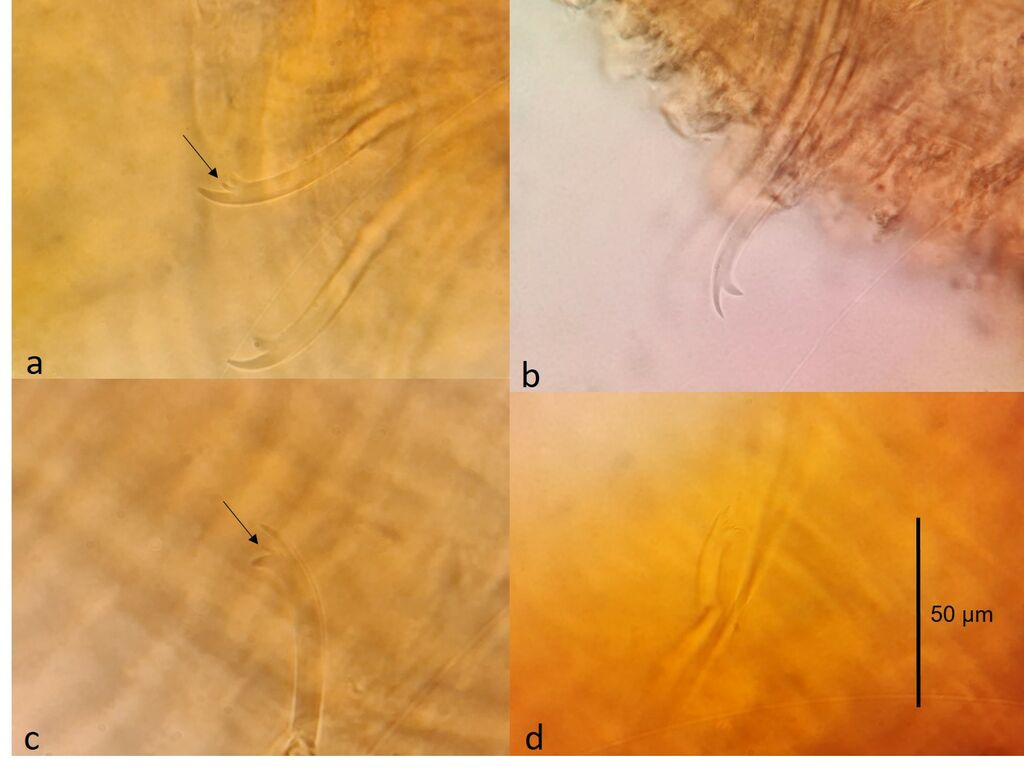
Photographs of **a-b** anterior ventral chaetae **c** posterior ventral chaeta and **d** pectinate chaetae of T.cf.americanus; the black arrow indicates the intermediate teeth.

**Figure 3.**
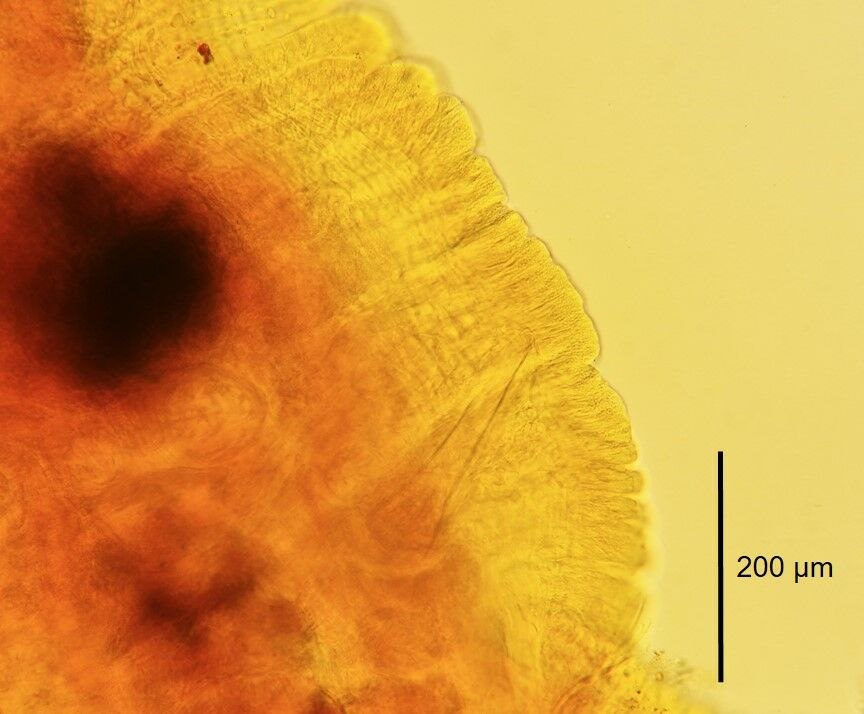
Photograph of the penis sheath of Tasserkidriluscf.americanus from Slovakia.

**Figure 4.**
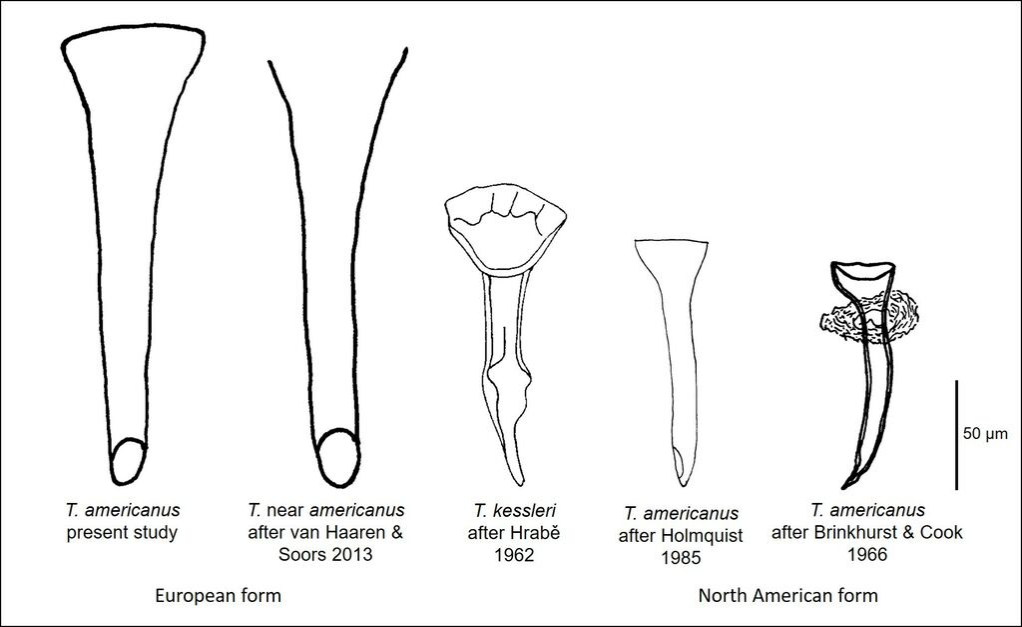
Drawings of the penial sheaths presenting the differences in shape and length between the North American and European populations of *T.* (cf.) *americanus* and *T.kessleri*. Drawings maintaining proportionality and shape of the penis sheath, based on photographs by van Haaren and Soors (2013) and Holmquist (1985), are free interpretations by the author.

Altogether, 63 specimens, of which 30 were sexually mature, were identified as Tasserkidriluscf.americanus. The density of the population reached 648 individuals per square metre. The species was diagnosed on the basis of its characteristic, external, morphological features. Ventral chaetae from segments II to V (or VI) have a longer upper tooth and several are equipped with an intermediate tooth, which can sometimes be doubled (Fig. 2a and b). From segment VII, the length of the upper and lower teeth equalises. Penial ventral chaetae in XI are thicker and shorter than anterior chaetae and in a reduced number or are completely absent. Posterior ventral chaetae have teeth equal in length, with the upper tooth 1 – 1.5 x thinner than the lower one and some of them may have an intermediate tooth (Fig. 2c). Hair chaetae present in anterior and posterior segments are up to 440 µm long. The species has pectinate chaetae similar to *Tubifextubifex* (Müller, 1774) (Fig. 2d). All chaetae are shorter in the posterior part of the body when compared with the anterior part. The length and number of chaetae are presented separately for each study describing the morphological features of the species in Table 2. Adult specimens have penis sheaths situated in segment XI (Fig. 3). Fig. 4 and Table 2 indicate the difference in shape and length of penis sheaths between populations of *T.americanus* and *T.kessleri*.

## Discussion

In Europe, frequent records of T.cf.americanus in streams have been documented from Belgium and The Netherlands (van Haaren and Soors 2013). The present finding of the species from Slovakia has reopened the discussion about the origin, distribution and taxonomy of the European population. The penis sheaths of individuals from Slovakia, Belgium and The Netherlands are proportionally larger and wider when compared to individuals occurring in North America, whereas no comparison can be made with the population from Russia due to lack of information. In Tubificinae, the proportions and specific shapes of penis sheaths are essential features for identification of many species of one same genus when the external features are similar. Therefore, the differences observed within *T.americanus* “forms” must be carefully considered. Actually, the issue of whether T.cf.americanus in Europe is the same as *T.americanus* in North America cannot be definitely resolved without genetic information for both forms. It is possible, in fact, that we are dealing with a cryptic species or subspecies. van Haaren and Soors (2013) preferred to name their material "Tasserkidrilusnearamericanus", because they did not have enough evidence on the identity of that species. Moreover, the authors mentioned that the record of *Ilyodrilustempletoni* (Southern, 1909) from the Estuary of the Elbe River (Germany), described by Pfannkuche (1977), was most likely the American form of *T.americanus*, based on the length and shape of the penis sheaths (Table 2). However, recent studies, which took place in the Elbe Estuary, did not confirm that the species really occurred there and neither did *I.templetoni* (see Wetzel et al. 2012).

The ecology of the discussed forms seems to be different as well, although the environmental requirements of North American populations of *T.americanus* showed a consistent pattern (Brinkhurst and Cook 1966, Hiltunen 1967). The species was found mostly in large, oligotrophic lakes, although Timm (2009), Spencer and Hudson (2003), and Soors et al. (2013) observed that the habitat of *T.americanus* includes rivers and coastal intertidal and subtidal waters as well. In contrast, the European populations of T.cf.americanus seem to be more tolerant to organic pollution, since they were found in eutrophic waters, marshes and, in the case of Slovakia, in the small channel with slow-flowing water near the swamp. van Haaren and Soors (2013) added that the European worms are tolerant to desiccation as well.

Another question that arises from the occurrence of T.cf.americanus in Slovakia is its origin: is it a non-indigenous species that was introduced from its natural area (North America), as stated by Soors et al. (2013), or is it native to Europe, but too rare to record? Moreover, van Haaren (2002) added that the identification of oligochaete species was not popular in Belgium in the past and, thus, the species may have been overlooked. This may also be the case of its current record in the channel in Slovakia, for which the latter has not undergone any research of its macroinvertebrate community or monitoring of its water quality by state institutions (Makovinská et al. 2015). Moreover, the Tešmak swamp belongs to The Poiplie Special Protected Area under the Ramsar Convention and Natura 2000. Therefore, the introduction of alien species by human activity is highly unlikely due to its protection status, although it cannot be ruled out. Even the Ipeľ River, which is a tributary of the Danube and flows near the swamp, cannot be considered a potential vector of the spreading of *T.americanus*, since it is a medium-sized river inappropriate for navigation and the species has not been documented there at all. Based on those arguments, the presence of T.cf.americanus in Slovakia remains inexplicable and requires further investigation.

## Supplementary Material

DCE863AD-2455-5EAC-87E6-CC3BF7BCDE0D 10.3897/BDJ.9.e72846.suppl1 Supplementary material 1 Data associated to T.cf.americanus occurrence in Slovakia Data type occurrences Brief description Excel table filled with required and recommended DwC data linked with the occurrence of Tasserkidriluscf.americanus in Slovakia File: oo_570563.xlsx https://binary.pensoft.net/file/570563 Igor Kokavec

## Figures and Tables

**Table 1.** Overview of the representatives of the genus *Tasserkidrilus*; ^1^disputable occurrence in the Lake Baikal (see Semernoy (2001)), ^2^ according to WoRMS (2021), ^3^ probable misidentification (see Semernoy (2001))

**Valid name**	**Synonymised names**	**Occurrence**	**Reference (except GBIF database)**
*Tasserkidrilusacapillatus* Finogenova, 1972	*Isochaetidesacapillatus* (Finogenova, 1972) *Tubifexacapillatus* Finogenova, 1972 *Tubifexkessleriacapillatus* Finogenova, 1972	Estonia, Russia, Baikal^1^, Kazakhstan, Azerbaijan, South Korea	Martin et al. (1999)
*Tasserkidrilusamericanus* (Brinkhurst & Cook, 1966)	*Tubifexkessleriamericanus* Brinkhurst & Cook, 1966	USA, Canada, Russia, Belgium, Germany?, Slovakia	Soors et al. (2013), van Haaren and Soors (2013)
*Tasserkidrilusbaicalensis* (Semernoy, 1982)	*Tubifexkessleribaicalensis* Semernoy, 1982	Russia (Baikal)	
*Tasserkidrilusheterodontus* Akinschina & Snimschikova, 1993		Russia (Baikal)	
*Tasserkidrilushrabei* (Sokolskaja, 1973)	*Tubifexhrabei* Sokolskaja, 1973	Russia (Kamchatka)	
*Tasserkidrilusinfundibuliferus* (Izosimov, 1972)	*Limnodrilusinfundibuliferus* Izosimov, 1972 *Tubifexkessleriinfundibuliferus*	Russia (Baikal)	
*Tasserkidriluskessleri* (Hrabě, 1962)	*Tubifexkessleri* Hrabě, 1962	USA, Russia (except Baikal), France, Switzerland, China	Jiang et al. (2010), Vivien et al. (2017)
*Tasserkidrilusmirandus* (Snimschikova, 1982)	*Tubifexmirandus* Snimschikova, 1982 *Tasserkidrilusheterodontus* Snimschikova & Akinschina, 1993 (unaccepted)^2^ *Tasserkidrilusrectitubifer* Snimschikova & Akinschina, 1993 (unaccepted)^2^ *Tasserkidrilustimmi* Snimschikova & Akinschina, 1993 (unaccepted)^2^	Russia (Baikal)	
*Tasserkidriluspenicraspedifer* (Semernoy, 1982)	*Tubifexpenicraspedifer* Semernoy, 1982 *Tubifexsolitarius* Semernoy, 1972 *Tasserkidrilussolitarius* (Semernoy, 1972)	Russia (Baikal)	
*Tasserkidrilusrectitubifer* Akinschina & Snimschikova, 1993		Russia (Baikal)	
*Tasserkidrilussuperiorensis* (Brinkhurst & Cook, 1966)	*Peloscolexsuperiorensis* Brinkhurst & Cook, 1966 *Tubifexsuperiorensis* (Brinkhurst & Cook, 1966)	USA, Hungary	Szitó (2005)
*Tasserkidrilustaediosus* (Čekanovskaja, 1975)	*Tubifextaediosus* Čekanovskaja, 1975	Russia (Baikal)	
*Tasserkidrilustimmi* Akinschina & Snimschikova, 1993		Russia (Baikal)	
*Tasserkidrilusvariabilis* (Semernoy, 1982)	*Tasserkidriluskesslerivariabilis* Semernoy, 1982 *Tubifexkesslerivariabilis* Semernoy, 1982 *Tasserkidrilus* (*Tubifex*, *Isochaetides*) *acapillatus*^3^ *Tasserkidrilussuperiorensis*^3^	Russia (Baikal)	

**Table 2.** Length (L) and number (Nr) of anterior and posterior () ventral chaetae (VS), dorsal chaetae (DS), hair chaetae (HS), chaetae in segment XI (XI) and length of penis sheaths (PSh) of populations of *T.kessleri* and *T.* (cf.) *americanus*, described in different countries; ^1^Hrabě (1962), ^2^Brinkhurst and Cook (1966), ^3^Holmquist (1985), ^4^van Haaren and Soors (2013), ^5^Pfannkuche (1977), ^6^present study; P - present in segments V, VI, VII, X, single and short (Brinkhurst and Jamieson 1971)

	**L_PSh** **(µ m)**	**Nr_VS**	**Nr_DS**	**Nr_HS**	**XI_VS**	**XI_DS**	**XI_HS**	**L_VS** **(µ m)**	**L_DS** **(µ m)**	**L_HS** **(µ m)**
***T.kessleri*^1^** (Russia)	140	3-5	3-5	P	2-3	1	0			P
***T.americanus*^2^** (USA: Great Lakes)	100	3-5	3-4	3-4						
***T.americanus*^3^** (USA: Alaska)	120	4-5 (2-3)	3-4	2-4 (1-2)	0	0-2	0-1			
**T.nearamericanus^4^** (Belgium)	160-285	3-4	3-4	3-5		1				-450
***T.americanus*^5^** (Germany; as *Ilyodrilustempletoni*)	100-160									
**T.cf.americanus^6^** (Slovakia)	179-259	3-4 (2-3)	2-4 (2-3)	3-4 (2-3)	1-2	0-2	0-2	110-161 (110-132)	112-143 (111-124)	235-432 (247-358)

## Hosting institution

Institute of Zoology, Slovak Academy of Sciences, Bratislava, Slovakia.

## References

[B7348717] Brinkhurst R. O., Cook D. G. (1966). Studies on the North American aquatic Oligochaeta III: Lumbriculidae and additional notes and records of other families. Proceedings of the Academy of Natural Sciences of Philadelphia.

[B7376494] Brinkhurst R. O., Jamieson B. G. M. (1971). Aquatic Oligochaeta of the World.

[B7376502] Secretariat GBIF *Tasserkidrilusamericanus* (Brinkhurst & Cook, 1966). https://www.gbif.org/occurrence/search?taxon_key=9479629.

[B7348903] Hiltunen J. K. (1967). Some oligochaetes from Lake Michigan. Transactions of the American Microscopical Society.

[B7348684] Holmquist C. (1985). A revision of the genera *Tubifex* Lamarck, *Ilyodrilus* Eisen, and *Potamothrix* Vejdovský & Mrázek (Oligochaeta, Tubificidae), with extensions to some connected genera. Zoologische Jahrbücher, Abteilung für Systematik, Ökologie und Geographie der Tiere.

[B7348726] Hrabě S. (1962). Oligochaeta limicola from Onega lake collected by Mr. B. M. Alexandrov. Spisy Přírodovědecké fakulty University J. E. Purkyně v Brne.

[B7348658] Hrabě S (1979). Vodní máloštetinatci (Oligochaeta) Československa. Acta Universitatis Carolinae - Biologica.

[B7350708] Jiang X. M., Xiong J., Qiu J. W., Wu J. M., Wang J. W., Xie Z. C. (2010). Structure of macroinvertebrate communities in relation to environmental variables in a subtropical Asian river system. International Review of Hydrobiology.

[B7348642] Kasprzak K. (1981). Skąposzczety wodne 1. Rodziny: Aeolosomatidae, Potamodrilidae, Naididae, Tubificidae, Dorydrilidae, Lumbriculidae, Haplotaxidae, Glossoscolecidae, Branchiobdellidae.

[B7348693] Makovinská J., Mišíková Elexová E., Rajczyková E., Plachá M., Kováč V., Fidlerová D., Ščerbáková S., Lešťáková M., Očadlík M., Velická Z., Horváthová G., Velegová V. (2015). Metodika monitorovania a hodnotenia vodných útvarov povrchových vôd Slovenska.

[B7350643] Martin P., Martens K., Goddeeris B. (1999). Oligochaeta from the abyssal zone of Lake Baikal (Siberia, Russia). Hydrobiologia.

[B7348921] Pfannkuche O. (1977). Ökologische und systematische Untersuchungen an naidomorphen Oligochaeten brackiger und limnischer Biotope.

[B7502757] Reynolds J. W., Wetzel M. J. Nomenclatura Oligochaetologica – A catalogue of names, descriptions and type specimens. Editio Secunda.. https://www.inhs.illinois.edu/people/mjwetzel/nomenoligo.

[B7350652] Semernoy V. P., Timoshkin O. A. (2001). Index of animal species inhabiting Lake Baikal and its catchment area.

[B7348772] Soors J., van Haaren T., Timm T., Speybroeck J. (2013). *Bratislaviadadayi* (Michaelsen, 1905) (Annelida: Clitellata: Naididae): a new non-indigenous species for Europe, and other non-native annelids in the Schelde estuary. Aquatic Invasions.

[B7348850] Spencer D. R., Hudson P. L. (2003). The Oligochaeta (Annelida, Clitellata) of the St. Lawrence Great Lakes region: an update. Journal of Great Lakes Research.

[B7350587] Szitó A. (2005). Earthworms (Annelida: Polychaeta and oligochaeta) of the river Tisza and its tributaries. Vegetation and Fauna of Tisza River Basin I..

[B7348675] Timm T. (2009). A guide to the freshwater Oligochaeta and Polychaeta of Northern and Central Europe. Lauterbornia.

[B7348817] van Haaren T. (2002). Eight species of aquatic Oligochaeta new for the Netherlands (Annelida). Nederlandse Faunistische Mededelingen.

[B7348634] van Haaren Ton, Soors Jan (2013). Aquatic oligochaetes of the Netherlands and Belgium.

[B7350558] Vivien Régis, Holzmann Maria, Werner Inge, Pawlowski Jan, Lafont Michel, Ferrari Benoit J. D. (2017). Cytochrome c oxidase barcodes for aquatic oligochaete identification: development of a Swiss reference database. PeerJ.

[B7348826] Wetzel M. A., von der Ohe P. C., Manz W., Koop J. H. E., Wahrendorf D-S (2012). The ecological quality status of the Elbe estuary. A comparative approach on different benthic biotic indices applied to a highly modified estuary. Ecological Indicators.

[B7350665] WoRMS *Tasserkidrilusmirandus* (Snimschikova, 1982). http://marinespecies.org/aphia.php?p=taxdetails&id=1040995.

